# The Dental News Letter

**Published:** 1848-01

**Authors:** 


*The Dental News Letter.*—We have the pleasure of noticing still another dental periodical, the Dental News Letter, to be published quarterly by Messrs. Jones, White & Co., New York, each number to contain sixteen pages. It is well printed and contains some very good articles.

There are now five periodicals devoted to the interests of the dental profession; namely, the Americal Journal and Library of Dental Science, the Dental Intelligencer, New York Dental Recorder, the Dental Register of the West, and the Dental News Letter. There is room enough for all, but we fear none will prove very profitable to the proprietors.

[*Balt. Ed.*

